# Importance of Thinking Locally for Mental Health: Data from Cross-Sectional Surveys Representing South East London and England

**DOI:** 10.1371/journal.pone.0048012

**Published:** 2012-12-12

**Authors:** Stephani L. Hatch, Charlotte Woodhead, Souci Frissa, Nicola T. Fear, Maria Verdecchia, Robert Stewart, Abraham Reichenberg, Craig Morgan, Paul Bebbington, Sally McManus, Traolach Brugha, Bwalya Kankulu, Jennifer L. Clark, Billy Gazard, Robert Medcalf, Matthew Hotopf, the SELCoH study team

**Affiliations:** 1 King's College London, Psychological Medicine, Institute of Psychiatry, London, United Kingdom; 2 King's College London, Academic Centre for Defence Mental Health, London, United Kingdom; 3 King's College London, Section of Epidemiology, Health Service and Population Research Department, Institute of Psychiatry, London, United Kingdom; 4 King's College London, Section of Social Psychiatry, Health Service and Population Research Department, Institute of Psychiatry, London, United Kingdom; 5 University College London, Department of Mental Health Sciences, London, United Kingdom; 6 National Centre for Social Research, London, United Kingdom; 7 Department of Health Sciences, New Academic Unit, Leicester General Hospital, Leicester, United Kingdom; Baylor College of Medicine, United States of America

## Abstract

**Background:**

Reliance on national figures may be underestimating the extent of mental ill health in urban communities. This study demonstrates the necessity for local information on common mental disorder (CMD) and substance use by comparing data from the South East London Community Health (SELCoH) study with those from a national study, the 2007 English Adult Psychiatric Morbidity Study (APMS).

**Methodology/Principal Findings:**

Data were used from two cross-sectional surveys, 1698 men and women residing in south London and 7403 men and women in England. The main outcome, CMD, was indicated by a score of 12 or above on the Revised Clinical Interview Schedule. Secondary outcomes included hazardous alcohol use and illicit drug use. SELCoH sample prevalence estimates of CMD were nearly twice that of the APMS England sample estimates. There was a four-fold greater proportion of depressive episode in the SELCoH sample than the APMS sample. The prevalence of hazardous alcohol use was higher in the national sample. Illicit drug use in the past year was higher in the SELCoH sample, with cannabis and cocaine the illicit drugs reported most frequently in both samples. In comparisons of the SELCoH sample with the APMS England sample and the APMS sample from the Greater London area in combined datasets, these differences remained after adjusting for socio-demographic and socioeconomic indicators for all outcomes.

**Conclusions/Significance:**

Local information for estimating the prevalence of CMD and substance use is essential for surveillance and service planning. There were similarities in the demographic and socioeconomic factors related to CMD and substance use across samples.

## Introduction

While national psychiatric epidemiological surveys are useful in setting macro-level policy agendas, combating inequalities in mental health requires knowledge generated from local data [1]. Epidemiological information allows high risk groups to be identified by services and also clarifies the adequacy of existing and planned service strategies. However, reliance on national figures may underestimate the extent of mental ill health in certain communities – for example in those with high levels of deprivation [2]–[3]. In this context, variations in the characteristics both of individual members of the population and of the area as a whole may engender concomitant variation in morbidity. Moreover, differences in prevalence of mental ill health and related needs may change in the face of changes in the distribution of economic resources and the availability of social services; in demography as a result of shifting migration patterns; and in the built environment, particularly in urban settings [4]–[9]. The impact of persistent, disabling and common mental disorders is most profound and costly in highly populated, urban communities [10]–[12]. However, data are lacking for such local communities, and there are methodological difficulties in extrapolating from national to local levels; for example national prevalence studies usually have insufficient sample size for robust analyses at the local level, and they may select only a proportion of the areas in their sampling frame [13]–[15]. Such difficulties are particularly acute if the major determinants of prevalence are characteristics of the area rather than of individual members of the population [13]. Thus, health planners need access to detailed local information in order to develop public mental health strategies. For this reason, we established the South East London Community Health Survey (SELCoH), which covers an inner-city population. Here we aimed to demonstrate the necessity for this level of information by comparing our data on the symptoms of common mental disorder (CMD) and the prevalence of common mental disorder (CMD) and substance use with those from a national study – the 2007 English Adult Psychiatric Morbidity Study (APMS). We hypothesise that the prevalence of CMD and substance use, including alcohol use, will be higher in the SELCoH sample in comparison to the APMS sample, and these differences will be explained by socio-demographic attributes and the socioeconomic status of the individual sample members.

## Methods

### Sample

The South East London Community Health (SELCoH) study is a local psychiatric and physical morbidity survey of 1698 adults, aged 16 years and over from 1075 randomly selected households in the South London boroughs of Southwark and Lambeth [16]. In the two boroughs, there is higher deprivation than the England average, but similar proportions of economically active and inactive residents in comparison to greater London [17]–[20]. The boroughs are also ethnically diverse, with a greater number of Black Caribbean residents but fewer South Asian residents than other areas of London [21]. The SELCoH sample resided in a community setting served by South London and Maudsley National Health Service Trust (SLaM) in the UK, and the partnership between King's College London and the SLaM NHS trust allows this and other research to inform and benefit clinical treatment.

Data were collected between June 2008 and December 2010 and the SELCoH study sampling strategy resulted in a 51.9% household participation rate and 71.9% participation within households. The study was conducted as a component of SLaM's NIHR Mental Health Biomedical Research Centre [16]. SELCoH aimed to provide updated local population data to inform the configuration of services.

The national data come from the Adult Psychiatric Morbidity Study (APMS) 2007 [22]. Data were collected in England between October 2006 and December 2007 from a random sample of private household residents aged 16 years and older. Using a multi-stage stratified probability sampling design [22], 7403 eligible individuals completed full interviews (57% of the total sampled).

SELCoH applied similar methods to the APMS [23], with a few notable exceptions. Both studies used the UK Small User Postcode Address File (PAF) for the sampling frames: this has near complete coverage of private households (defined as one person or group of people who have the accommodation as their only or main residence and for groups who either share at least one meal a day or share the living area). Trained interviewers conducted interviews in participants' homes and administered structured assessments using laptops. The same measures were used for the principal outcomes, and many of the questions on demographic variables within SELCoH were based upon APMS methodology. Differences in methods included attempts in the SELCoH sample to interview all adults aged 16 years and over in each eligible household, whereas the APMS sample randomly selected one adult, aged 16 years and over to be interviewed in each eligible household using the Kish grid method. In addition, the APMS sample was stratified by region (Strategic Health Authorities) and manual and non-manual social class [22], whereas the SELCoH sample was stratified by borough.

### Ethics statement

Ethical approval for the SELCoH study was received from the King's College London research ethics committee for non-clinical research populations; reference CREC/07/08-152. Ethical approval for APMS 2007 was obtained from the Royal Free Hospital and Medical School Research Ethics Committee, one of the Research Ethics Committees of the National Research Ethics Service for non-clinical populations.

### Measures

#### Common mental disorder

Common mental disorder (CMD) in both samples was assessed by the Revised Clinical Interview Schedule (CIS-R) [24] - a structured interview that asks about 14 symptom domains (using skips to allow asymptomatic individuals to answer a minimum of 28 questions): fatigue, sleep problems, irritability, worry, depression, depressive ideas, anxiety, obsessions, subjective memory and concentration, somatic symptoms, compulsions, phobias, physical health worries and panic. A total CIS-R score of 12 or more is conventionally used to indicate the overall presence of CMD, with a total score of 18 or more that denotes a level of symptoms that are likely to require treatment. The CIS-R also provides ICD-10 diagnoses for six mental disorders through a standard algorithm (depressive episode, generalised anxiety disorder, panic disorder, phobias, obsessive-compulsive disorder and mixed anxiety and depressive disorder). Mixed anxiety and depressive disorder is a residual category, covering cases that have a total CIS-R score of 12 or more, but do not meet the specific criteria for the other five disorders. As such, it tends to be less severe, particularly in comparison to depressive episode, and less likely to be associated with treatment seeking and treatment receipt [25]–[26].

#### Substance use

In both samples, hazardous alcohol use was assessed by the Alcohol Use Disorders Identification Test (AUDIT) [27], developed by the World Health Organization, and comprising ten questions relating to alcohol consumption, symptoms of alcohol dependence and problems related to alcohol abuse within the last 12 months. Each item is scored 0–4 with a summed overall score ranging from 0–40. For this analysis, an AUDIT score of 8 or more has been used to define hazardous drinking; AUDIT scores were also recoded into four groups: an AUDIT score of 0 has been used to define non-drinkers, 1 to 7 indicated moderate drinkers, 8 to 15 defined hazardous alcohol use, and a score of 16 or more defined hazardous alcohol use that is harmful to health. Participants reported illicit drug use in the past year for the following drugs in both samples: cannabis, amphetamines, cocaine, crack, ecstasy, LSD, tranquilliser and heroin. *Any drug use* in the past year referred to use of at least one drug in the past year, while concurrent *poly drug use* (in the same time period) [28] was defined as the use of 2 or more drugs.

#### Socio-demographic and socio-economic factors

The following socio-demographic and socioeconomic indicators available in both samples were included: gender; the ethnic group categories of White British, Black Caribbean, Black African, South Asian or Other; age (years) both as a continuous variable and in the following categories: 16–24, 25–34, 35–44, 45–54, 55–64, and 65 and over; marital status; education level categorised as: no qualifications, qualifications up to GCSE or Ordinary Level, Advanced Level, and degree level or above; occupational social class (non-manual vs. manual); employment status categorised as: paid employment, unemployed, and economically inactive (i.e., student, permanent sick/disabled, temporary sick, retired, looking after the home and children); and housing tenure categorised as: own/mortgage, rented and rent free.

### Analysis

Analyses were conducted in STATA 11 [29]. We used survey commands (svy) for estimates of prevalence and associations where appropriate to generate robust standard errors.

All analyses of SELCoH data accounted for clustering by household inherent in the study design and weighted for within household non-response, comparing all eligible household members (i.e., 16 years or older) by gender and age. As previously reported [16], the sample was similar to the most recent UK Census information in 2001 with regards to demographic and socioeconomic indicators for the catchment area under study, with the exception of the sample being slightly younger and having more students within the economically inactive group. However, there were no differences in the distribution by age across categories in comparisons of the SELCoH and APMS samples. In reference to the proportion of students, there were more students identified in the SELCoH sample (12.5%) in comparison to the APMS sample (3.3%); however, student status is likely to be underestimated in the APMS sample because the survey only inquired about student status as a response to a question asking about the main reason for being currently out of work.

All analyses of APMS data accounted for weighting, clustering and stratification built into the survey design. Weighting in the APMS data accounted for clusters by postcode sectors, stratification based on socio-economic status within regional areas and non-response based on differences between the sample and the mid-census estimates [22]. Analysis was conducted for those with complete data for all variables. We report the unweighted frequencies, and applied Pearson's X^2^ tests with Rao & Scott second-order corrections with 95 percent confidence intervals for categorical outcomes. Odds ratios (OR) with 95 percent confidence intervals (CI) were calculated for the associations of categorical outcomes with socio-demographic and socio-economic indicators. Models adjusted for gender and age in years are presented for all logistic regression models. To examine whether or not identified differences will be explained by socio-demographic attributes and the socioeconomic status of the individual sample members, data were combined to make direct comparisons across samples in fully adjusted multivariable logistic regression models for fully adjusted models. The definition of social class in SELCoH excludes participants without a current occupation; thus, social class is not included in the fully adjusted models for the combined dataset.

## Results

### Sample characteristics

Compared with the national survey data, the SELCoH sample had an over-representation of women, the youngest participants, Black Caribbean and Black African groups, never-married and divorced/separated groups, those with higher education levels, and the non-manual and the unemployed groups (Table 1).

**Table 1:** Socio-demographic characteristics of the South East London Community Health (SELCoH) and Adult Psychiatric Morbidity Study (APMS) 2007 England samples.

		SELCoH	APMS 2007
**Total**		1698	7403
**Gender**	Female	959 (66.7)	4206 (51.4)
	Male	739 (33.3)	3197 (48.6)
**Ethnic group**	White British	1051 (63.5)	6499 (85.1)
	Black-Caribbean	143 (8.7)	104 (1.5)
	Black-African	234 (13.2)	78 (1.5)
	Asian	63 (3.5)	258 (5.0)
	Other	205 (11.2)	414 (6.9)
**Age (years)**	16–24	356 (18.2)	568 (14.2)
	25–39	572 (28.6)	1744 (26.1)
	40–54	432 (24.1)	1834 (25.9)
	55–64	163 (13.3)	1279 (14.8)
	65+	175 (15.9)	1978 (19.0)
**Marital status**	Never married	678 (35.7)	1428 (22.7)
	Married/cohabiting	786 (46.4)	4133 (62.9)
	Divorced/separated	181 (12.6)	893 (7.5)
	Widowed	53 (5.3)	949 (7.0)
**Education levels**	No qualifications	228 (16.9)	2278 (26.2)
	Up to GCSE level	332 (20.1)	2103 (30.9)
	Advanced level	426 (23.7)	938 (15.1)
	Higher degree or above	693 (39.3)	1916 (27.8)
**Social class** a	Non-manual	703 (73.4)	4277 (60.8)
	Manual	244 (26.6)	2732 (39.2)
**Employment status**	Paid employment	921 (51.2)	3964 (60.4)
	Unemployed	170 (9.3)	164 (2.9)
	Economically inactive	598 (39.5)	3250 (36.7)
**Housing tenure**	Own/mortgage	525 (32.4)	5143 (69.8)
	Rented	1058 (61.7)	2077 (28.3)
	Rent free	112 (5.8)	117 (1.9)

Values are numbers (percentages) of respondents; weighted percentages to account for survey design; frequencies are unweighted and may not add up due to missing values.

a Social class is based on occupation and participants without a current occupation were excluded in both samples.

### Prevalence of CMD symptoms and diagnostic categories by gender

Table 2 compares the one-week prevalences and 95% confidence intervals (CI) for the distributions of CIS-R scores and psychiatric disorders across the two samples. A greater proportion of SELCoH sample met the criteria for CMD than the APMS sample. The differences in proportions was present for those with CIS-R scores at 12 to 17 and 18 and above, the latter denoting a level of symptoms that are likely to require treatment [17]. Overall, psychiatric diagnoses identified using CIS-R were significantly more common in the SELCoH sample than in the APMS sample (p<0.001). There was a striking discrepancy in the prevalence of depressive episode. This, the most symptomatically severe of the common mental disorders covered in the CIS-R, was four times more frequent in the SELCoH sample than in the national survey and was indeed the most prevalent mental disorder in the SELCoH participants. In contrast, the most common psychiatric diagnosis in the APMS sample was the residual diagnosis of mixed anxiety and depressive disorder, and there was no difference in the prevalence of this condition between the SELCoH and APMS samples. Obsessive compulsive disorder and panic disorder were the least common psychiatric diagnoses in both samples, but the prevalence of these disorders was greater in the APMS sample (p<0.001 and p = 0.03, respectively).

**Table 2:** Comparisons of prevalence estimates of common mental conditions and psychiatric diagnoses by gender.

	Total			Women			Men		
	SELCoH	APMS 2007		SELCoH	APMS 2007		SELCoH	APMS 2007	
	% (95%CI)	% (95%CI)	P value of difference	% (95%CI)	% (95%CI)	P value of difference	% (95%CI)	% (95%CI)	P value of difference
**CIS-R scores**									
CMD ‘case’ (12+)	24.2 (21.9–26.5)	15.1 (14.1–16.0)	<0.001	27.3 (24.3–30.3)	18.4 (17.0–19.8)	<0.001	17.9 (15.0–20.9)	11.6 (10.3–12.8)	<0.001
0–5	54.9 (52.3–57.6)	68.1 (66.7–69.4)	<0.001	50.3 (46.9–53.6)	62.3 (60.5–64.2)	<0.001	64.3 (60.7–67.9)	74.2 (72.3–75.9)	<0.001
6–11	20.9 (18.8–22.9)	16.9 (15.8–17.9)		22.4 (19.7–25.1)	19.3 (17.9–20.7)		17.7 (14.8–20.5)	14.3 (12.8–15.7)	
12–17	11.7 (10.1–13.4)	7.5 (6.8–8.3)		12.5 (10.4–14.6)	9.1 (8.0–10.2)		10.2 (7.9–12.5)	5.9 (4.9–6.8)	
≥18	12.4 (10.7–14.2)	7.5 (6.9–8.2)		14.8 (12.5–17.1)	9.3 (8.4–10.2)		7.8 (5.7–9.8)	5.7 (4.8–6.5)	
**Primary diagnosis**									
Mixed anxiety and depressive disorder	7.7 (6.4–9.1)	8.4 (7.7–9.2)	0.4	8.9 (7.1–10.8)	10.3 (9.2–11.5)	0.2	5.3 (3.7–6.9)	6.4 (5.6–7.4)	0.3
Depressive episode	11.9 (10.2–13.6)	2.9 (2.6–3.4)	<0.001	13.7 (11.4–15.9)	3.5 (3.0–4.1)	<0.001	8.4 (6.3–10.5)	2.4 (1.9–3.1)	<0.001
Generalised anxiety disorder	4.4 (3.4–5.5)	4.4 (3.9–4.9)	0.9	4.8 (3.4–6.3)	5.3 (4.6–6.1)	0.6	3.7 (2.3–5.1)	3.4 (2.8–4.2)	0.7
Obsessive-compulsive disorder	0.1 (0.0–0.3)	1.1 (0.9–1.4)	<0.001	0.1 (0.01–0.2)	1.3 (0.9–1.8)	<0.001	0.2 (0.2–0.6)	0.9 (0.6–1.4)	0.1
Phobias	2.8 (1.9–3.6)	2.0 (1.7–2.4)	0.1	2.7 (1.7–3.7)	2.7 (2.2–3.4)	0.9	2.9 (1.7–4.2)	1.3 (0.9–1.8)	0.002
Panic disorder	0.5 (0.1–0.9)	1.1 (0.9–1.4)	0.03	0.6 (0.1–1.1)	1.3 (0.9–1.7)	0.1	0.2 (0.1–0.6)	1.0 (0.7–1.5)	0.03

Weighted percentages to account for survey design.

**Figure 1:**
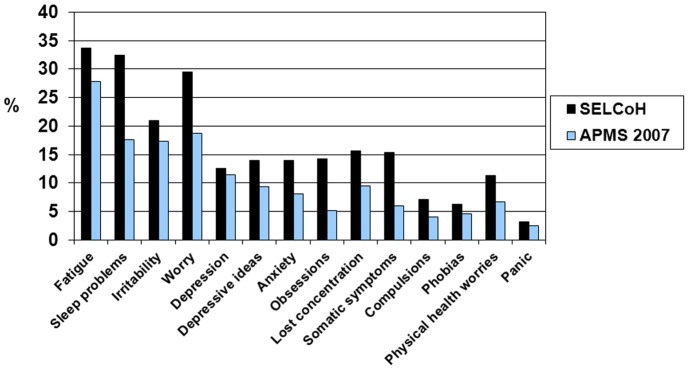
Comparisons of weighted prevalence estimates of Revised Clinical Interview Schedule symptom scores (≥2).

The prevalence estimates for the CIS-R symptoms are shown in Figure 1. In both samples, fatigue was the most commonly reported symptom (33.7% in the SELCoH sample and 27.8% in the APMS sample), followed by sleep problems (32.5% in the SELCoH sample and 17.6% in the APMS sample) and worry (29.5% in the SELCoH sample and 18.7% in the APMS sample). Panic was the least common symptom reported (3.2% in the SELCoH sample and 2.5% in the APMS sample). An increase in symptom reporting was generally observed in the SELCoH sample; however, despite the disparity in the prevalence of a depressive episode diagnosis, depressed mood was one of the symptoms with a similar prevalence in the two surveys.

### Socio-demographic and socio-economic factors and CMD

Table 3 compares the one-week prevalence for CMD by socio-demographic and socio-economic indicators across samples. There was a higher prevalence of CMD in the SELCoH sample within each stratum of all indicators, with the exception of comparisons between those in the Black African ethnic group. In general, there were similar associations between the majority of socio-demographic and socioeconomic indicators and CMD, with the exception of ethnicity and social class. There was no association between ethnicity and CMD in the SELCoH sample, although there was a trend towards lower prevalence of CMD in the Black African group than other groups (as previously noted in this area of south London [30]). In contrast, people in the Black African group in the APMS sample were more likely to meet the criteria for CMD than the White British group.

**Table 3:** One-week prevalence and adjusted odds ratios for common mental disorder (Revised Clinical Interview Schedule score ≥12) by socio-demographic and socio-economic indicators.

		SELCoH		APMS England 2007	
		% (95%CI)	Adjusted odds ratio^a^ (95%CI), p-value	% (95%CI)	Adjusted odds ratio^a^ (95%CI), p-value
**Gender**	Female	27.3 (24.3–30.2)	1.7 (1.4–2.2), p<0.001	18.4 (17.0–19.8)	1.8 (1.5–2.1), p<0.001
	Male	17.9 (15.0–20.8)	1.0	11.6 (10.3–12.9)	1.0
**Ethnic group**	White British	24.3 (21.5–27.3)	1.0	14.5 (13.5–15.5)	1.0
	Black-Caribbean	31.0 (22.3–39.8)	1.4 (0.9–2.1), NS	17.1 (9.9–27.9)	1.1 (0.5–2.2), NS
	Black-African	19.5 (13.8–25.1)	0.7 (0.5–1.1), NS	23.1 (16.1–31.9)	1.6 (1.0–2.6),p = 0.04
	Asian	24.9 (15.9–33.9)	1.0 (0.6–1.7), NS	13.6 (9.8–18.6)	0.9 (0.6–1.3), NS
	Other	23.0 (16.8–29.3)	0.9 (0.6–1.3), NS	18.3 (14.8–22.4)	1.2 (0.9–1.6), NS
**Age (years)**	16–24	25.1 (20.2–30.0)	1.0	16.4 (13.4–19.9)	1.0
	25–39	21.9 (18.3–25.6)	0.8 (0.6–1.1), NS	16.9 (15.1–19.0)	1.0 (0.8–1.4), NS
	40–54	29.5 (24.8–34.1)	1.2 (0.9–1.7), NS	17.6 (15.8–19.5)	1.1 (0.8–1.4), NS
	55–64	25.4 (18.2–32.5)	1.0 (0.6–1.6), NS	13.2 (11.3–15.3)	0.8 (0.6–1.0), NS
	65+	18.3 (12.3–24.3)	0.7 (0.4–1.1), NS	9.5 (8.1–11.1)	0.5 (0.4–0.7), p<0.001
**Marital status**	Never married	26.5 (22.9–30.2)	1.0	18.2 (16.1–20.5)	1.0
	Married/cohabiting	19.8 (16.8–22.9)	0.7 (0.5–0.9), p = 0.04	12.8 (11.8–13.9)	0.8 (0.7–1.0), p = 0.04
	Divorced/separated	32.2 (25.1–39.4)	1.3 (0.9–2.0), NS	24.6 (21.7–27.9)	1.8 (1.4–2.3), p<0.001
	Widowed	27.1 (14.9–39.4)	1.1 (0.5–2.3), NS	14.8 (12.3–17.7)	1.2 (0.9–1.6), NS
**Education levels**	No qualifications	25.7 (19.8–31.6)	1.7 (1.1–2.5), p = 0.01	17.4 (15.5–19.4)	2.0 (1.6–2.5), p<0.001
	Up to GCSE level	30.5 (25.2–35.8)	1.9 (1.4–2.6), p<0.001	16.3 (14.7–18.1)	1.5 (1.2–1.8), p<0.001
	Advanced level	25.6 (21.2–29.9)	1.4 (1.0–1.9), p = 0.02	14.4 (12.1–17.0)	1.3 (1.0–1.6), NS
	Higher degree or above	19.2 (16.1–22.3)	1.0	11.3 (9.9–12.9)	1.0
**Social class**	Non-manual	19.9 (16.7–23.1)	1.0	13.6 (12.6–14.7)	1.0
	Manual	22.2 (16.4–28.0)	1.2 (0.8–1.8), NS	16.5 (15.0–18.2)	1.4 (1.2–1.6), p<0.001
**Employment status**	Paid employment	20.1 (17.3–22.9)	1.0	12.6 (11.5–13.8)	1.0
	Unemployed	35.4 (27.9–43.0)	2.2 (1.5–3.2), p<0.001	27.3 (19.4–37.1)	2.4 (1.5–3.7), p<0.001
	Economically inactive	26.9 (23.1–30.6)	1.5 (1.1–1.9), p = 0.003	18.2 (16.6–19.8)	1.9 (1.6–2.2), p<0.001
**Housing tenure**	Own/mortgage	18.9 (15.1–22.7)	1.0	11.8 (10.8–12.9)	1.0
	Rented	27.9 (24.8–30.9)	1.6 (1.2–2.1), p = 0.002	22.5 (20.5–24.3)	2.0 (1.7–2.4), p<0.001
	Rent free	14.2 (7.4–21.1)	0.6 (0.4–1.2), NS	16.9 (10.3–26.5)	1.5 (0.8–2.6), NS

NS = non-significant; Weighted percentages to account for survey design;

a . Model adjusted for age and gender.

### Prevalence of substance use by gender

As shown in Table 4, there was a higher prevalence of hazardous alcohol use in the APMS sample than in the SELCoH sample. In both samples, men reported more hazardous alcohol use than women (p<0.001). In contrast to alcohol use, there was a two-fold higher prevalence of any illicit drug use in the past year in the SELCoH sample compared to the APMS sample. Further, there was a higher prevalence of concurrent poly drug use in the past year in the SELCoH sample. In both samples, cannabis was the most commonly used drug in the past year, but reported by a greater proportion of the SELCoH sample. Cocaine was the second most commonly reported drug in the past year in both samples. In both samples, men reported more cannabis and cocaine use in the past year than women.

**Table 4:** Comparisons of prevalence estimates for substance use by gender between SELCoH and APMS England 2007.

	All			Women			Men		
	SELCoH	APMS 2007		SELCoH	APMS 2007		SELCoH	APMS 2007	
	% (95%CI)	% (95%CI)	P value	% (95%CI)	% (95%CI)	P value	% (95%CI)	% (95%CI)	P value
**Alcohol Use**									
Score 0	24.5 (22.0–27.0)	17.3 (16.2–18.4)		27.4 (24.3–30.5)	22.0 (20.6–23.4)		18.6 (15.4–21.8)	12.3 (10.9–13.8)	
Score 1–7	58.7 (56.0–61.4)	58.6 (57.3–59.9)		60.4 (57.1–63.6)	62.4 (60.6–64.1)		55.4 (51.5–59.3)	54.6 (52.6–56.6)	
Score 8–15	13.9 (12.1–15.6)	20.4 (19.3–21.6)		10.4 (8.5–12.4)	13.8 (12.6–15.0)		20.7 (17.7–23.8)	27.4 (25.6–29.2)	
Score 16–40	2.9 (2.1–3.7)	3.8 (3.3–4.4)		1.8 (1.0–2.6)	1.9 (1.5–2.5)		5.2 (3.6–6.9)	5.8 (4.8–6.8)	
Hazardous alcohol use (Score 8+)	17.5 (15.5–19.5)	24.2 (22.9–25.4)	<0.001	12.9 (10.8–15.1)	15.7 (14.4–16.9)	= 0.04	26.7 (23.2–30.1)	33.2 (31.3–35.1)	= 0.002
**Past Year Drug Use**									
Cannabis	15.2 (15.9–20.1)	7.6 (6.7–8.4)		12.1 (9.9–14.3)	5.0 (4.2–6.0)		21.4 (18.4–24.5)	10.1 (8.8–11.5)	
Amphetamine	1.4 (0.9–1.9)	0.7 (0.4–1.0)		0.8 (0.3–1.4)	0.4 (0.2–0.7)		2.5 (1.4–3.5)	1.1 (0.7–1.6)	
Cocaine	6.3 (5.1–7.5)	2.5 (2.1–2.9)		4.2 (2.9–5.5)	1.4 (1.0–1.9)		10.4 (8.2–12.7)	3.6 (2.9–4.5)	
Crack	0.2 (0.0–0.4)	0.2 (0.1–0.4)		0.1 (0.1–0.2)	–		0.4 (0.0–0.8)	0.4 (0.2–0.8)	
Ecstasy	3.8 (2.8–4.8)	1.2 (0.9–1.5)		2.6 (1.5–3.6)	0.6 (0.4–0.8)		6.3 (4.6–7.9)	1.9 (1.4–2.5)	
LSD	0.7 (0.3–1.0)	0.2 (0.1–0.3)		0.5 (0.1–0.9)	0.0 (0.0–0.2)		0.9 (0.3–1.6)	0.4 (0.2–0.7)	
Tranquilliser	1.7 (1.0–2.4)	0.7 (0.5–0.9)		1.9 (1.0–2.8)	0.5 (0.5–0.8)		1.2 (0.5–1.9)	0.9 (0.6–1.3)	
Heroin	0.1 (0.0–0.3)	0.2 (0.1–0.3)		0.1 (0.1–0.2)	–		0.3 (0.1–0.6)	0.3 (0.2–0.6)	
Any drugs (≥1 drugs)	18.1 (16.1–20.2)	8.9 (8.1–9.8)	<0.001	14.3 (11.9–16.6)	6.3 (5.5–7.3)	<0.001	25.8 (22.5–29.2)	11.6 (10.3–13.1)	<0.001
Poly drug use (≥2 drugs)	6.3 (5.1–7.5)	2.4 (2.0–2.8)	<0.001	4.5 (3.2–5.9)	1.1 (0.8–1.5)	<0.001	9.7 (7.6–1.9)	3.4 (3.0–4.6)	<0.001

Weighted percentages to account for survey design.

### Substance use by socio-demographic and socio-economic factors

Tables 5 and 6 compare the prevalence and confidence intervals for hazardous alcohol use and illicit drug use by socio-demographic and socio-economic indicators. There was a higher prevalence of hazardous alcohol use and a lower prevalence of illicit drug use in the APMS sample across all strata of indicators, except for a lower proportion of hazardous alcohol use in the Asian ethnic group in the APMS sample. With few exceptions, there were similar associations between the socio-demographic indicators with both substance use outcomes across samples. However, those who were married or cohabitating and widowed in the APMS sample were less likely to report hazardous alcohol use in comparison to those in the never married group. In addition, those in the Black Caribbean group in the APMS sample were more likely to report illicit drug use than those in the White ethnic group, but there was no difference between the two ethnic groups in the SELCoH sample. Among the socio-economic indicators, those with a higher education level and in a non-manual social class were more likely to report hazardous alcohol use in the SELCoH sample, but there was no difference in these respects in the APMS sample. There was no association between employments status in the SELCoH sample, but those were economically inactive in the APMS sample were less likely to report hazardous alcohol use. There were also greater odds of illicit drug use among those in manual social class and those in rented accommodation in the APMS sample, but not in the SELCoH sample.

**Table 5:** Prevalence estimates and adjusted odds ratios for hazardous alcohol use by socio-demographic and socio-economic indicators.

		SELCoH		APMS England 2007	
		% (95%CI)	Adjusted odds ratio^a^ (95%CI), p-value	% (95%CI)	Adjusted odds ratio^a^ (95%CI), p-value
**Gender**	Female	11.3 (9.2–13.3)	0.4 (0.3–0.5), p<0.001	15.7 (14.4–16.9)	0.4 (0.3–0.4), p<0.001
	Male	24.6 (21.2–27.9)	1.0	33.2 (31.3–35.1)	1.0
**Ethnic group**	White British	20.5 (17.9–23.2)	1.0	25.5 (24.2–26.8)	1.0
	Black-Caribbean	3.8 (0.8–6.8)	0.1 (0.1–0.3), p<0.001	17.5 (8.6–26.4)	0.6 (0.3–1.1), NS
	Black-African	3.4 (1.2–5.7)	0.1 (0.0–0.2), p<0.001	8.2 (0.7–15.7)	0.2 (0.1–0.5), p = 0.001
	Asian	16.3 (6.9–25.6)	0.6 (0.3–1.3), NS	8.7 (5.1–12.4)	0.2 (0.1–0.3), p<0.001
	Other	11.8 (7.2–16.4)	0.4 (0.2–0.6), p<0.001	23.9 (19.0–28.8)	0.7 (0.6–1.0), NS
**Age (years)**	16–24	22.7 (17.8–27.7)	1.0	33.2 (32.6–41.9)	1.0
	25–39	22.5 (18.8–26.2)	1.0 (0.7–1.5), NS	29.3 (26.9–31.7)	0.7 (0.5–0.9), p = 0.004
	40–54	13.4 (10.1–16.7)	0.5 (0.4–0.8), p = 0.003	23.2 (21.1–25.3)	0.5 (0.4–0.6), p<0.001
	55–64	10.2 (5.6–14.8)	0.4 (0.2–0.7), p<0.001	19.3 (16.9–21.6)	0.4 (0.3–0.5), p<0.001
	65+	3.3 (0.8–5.7)	0.1 (0.0–0.3), p<0.001	12.4 (10.8–14.0)	0.2 (0.2–0.3), p<0.001
**Marital status**	Never married	21.4 (17.9–24.9)	1.0	36.7 (33.5–39.8)	1.0
	Married/cohabiting	12.9 (10.5–15.3)	0.9 (0.6–1.2), NS	21.3 (20.0–22.7)	0.7 (0.6–0.8), p<0.001
	Divorced/separated	14.1 (9.2–19.0)	1.5 (0.9–2.4), NS	24.2 (21.1–27.3)	1.0 (0.8–1.3), NS
	Widowed	4.8 (0.7–10.2)	1.0 (0.3–3.5), NS	8.6 (6.7–10.5)	0.5 (0.4–0.8), p<0.001
**Education levels**	No qualifications	9.9 (5.9–13.9)	0.7 (0.4–1.2), NS	17.4 (15.5–19.4)	0.9 (0.7–1.1), NS
	Up to GCSE level	9.3 (6.4–12.2)	0.3 (0.2–0.5), p<0.001	24.1 (21.9–26.2)	0.9 (0.7–1.0), NS
	Advanced level	15.6 (11.9–19.3)	0.5 (0.4–0.8), p<0.001	32.9 (29.4–36.5)	1.2 (0.9–1.4), NS
	Higher degree or above	21.7 (18.5–24.9)	1.0	26.0 (23.9–28.2)	1.0
**Social class**	Non-manual	19.5 (16.5–22.6)	1.0	22.9 (21.4–24.3)	1.0
	Manual	9.8 (5.9–13.6)	0.5 (0.3–0.7), p = 0.002	26.8 (24.8–28.8)	1.1 (0.9–1.2), NS
**Employment status**	Paid employment	17.0 (14.5–19.6)	1.0	28.8 (27.1–30.4)	1.0
	Unemployed	20.6 (14.2–27.0)	1.2 (0.7–1.8), NS	33.4 (24.8–41.9)	0.9 (0.6–1.4), NS
	Economically inactive	12.9 (9.9–15.9)	0.9 (0.6–1.2), NS	15.8 (14.3–17.3)	0.7 (0.6–0.9), p<0.001
**Housing tenure**	Own/mortgage	13.9 (10.8–16.9)	1.0	23.3 (21.9–24.8)	1.0
	Rented	16.4 (13.9–18.9)	0.9 (0.7–1.4), NS	25.8 (23.6–28.3)	0.9 (0.8–1.1), NS
	Rent free	18.3 (10.1–26.5)	0.7 (0.4–1.4), NS	32.6 (22.6–44.4)	1.3 (0.7–2.2), NS

NS = non-significant; Weighted percentages to account for survey design;

a . Model adjusted for age and gender.

**Table 6:** Comparisons of prevalence estimates and adjusted odds ratios for illicit drug use by socio-demographic and socio-economic indicators.

		SELCoH		APMS England 2007	
		% (95%CI)	Adjusted odds ratio^a^ (95%CI), p-value	% (95%CI)	Adjusted odds ratio^a^ (95%CI), p-value
**Sample**	SELCoH				
	APMS 2007				
**Gender**	Female	14.3 (11.9–16.6)	0.5 (0.4–0.6), p<0.001	6.3 (5.5–7.3)	0.5 (0.4–0.6), p<0.001
	Male	25.8 (22.5–29.2)	1.0	11.6 (10.3–13.1)	1.0
**Ethnic group**	White British	24.3 (21.5–27.3)	1.0	8.5 (7.6–9.3)	1.0
	Black-Caribbean	31.0 (22.3–39.8)	0.9 (0.5–1.5), NS	19.0 (10.1–27.9)	2.7 (1.4–5.2), p = 0.003
	Black-African	19.5 (13.8–25.1)	0.1 (0.1–0.2), p<0.001	8.9 (1.0–16.8)	0.6 (0.2–1.6), NS
	Asian	24.9 (15.9–33.9)	0.2 (0.1–0.4), p<0.001	3.6 (0.9–6.3)	0.2 (0.1–0.5), p<0.001
	Other	23.0 (16.8–29.3)	0.6 (0.4–0.9), p = 0.02	16.0 (11.6–20.4)	1.5 (1.0–2.1), p = 0.03
**Age (years)**	16–24	32.5 (26.7–38.3)	1.0	23.7 (19.8–27.5)	1.0
	25–39	25.2 (21.3–29.1)	0.7 (0.5–0.9), p = 0.05	14.9 (13.0–16.9)	0.6 (0.4–0.7), p<0.001
	40–54	14.6 (11.1–18.1)	0.4 (0.2–0.5), p<0.001	4.5 (3.5–5.5)	0.1 (0.1–0.2), p<0.001
	55–64	8.9 (4.6–13.3)	0.2 (0.1–0.4), p<0.001	2.1 (1.2–2.9)	0.1 (0.04–0.1), p<0.001
	65+	1.8 (0.3–3.9)	0.04 (0.0–0.1), p<0.001	0.8 (0.4–1.3)	0.03(0.02–0.05), p<0.001
**Marital status**	Never married	30.5 (26.5–34.5)	1.0	21.8 (19.2–24.4)	1.0
	Married/cohabiting	11.4 (9.0–13.8)	0.5 (0.4–0.7), p<0.001	4.9 (4.1–5.6)	0.5 (0.4–0.7), p<0.001
	Divorced/separated	14.8 (9.8–19.8)	1.1 (0.6–1.7), NS	10.0 (7.5–12.5)	1.8 (1.2–2.6), p = 0.002
	Widowed	2.0 (0.8–4.9)	0.3 (0.1–1.4), NS	1.5 (0.5–2.5)	1.1 (0.5–2.3), NS
**Education levels**	No qualifications	9.7 (6.1–13.3)	1.0 (0.6–1.6), NS	5.3 (4.0–6.6)	1.2 (0.8–1.7), NS
	Up to GCSE level	19.3 (14.8–23.7)	0.9 (0.7–1.4), NS	10.2 (8.5–11.9)	0.9 (0.7–1.3), NS
	Advanced level	21.6 (17.2–25.9)	0.9 (0.6–1.3), NS	13.7 (10.9–16.4)	1.1 (0.8–1.6), NS
	Higher degree or above	19.4 (16.2–22.5)	1.0	8.5 (6.8–10.2)	1.0
**Social class**	Non-manual	22.3 (18.9–25.7)	1.0	7.4 (6.4–8.4)	1.0
	Manual	16.5 (11.7–21.3)	0.8 (0.5–1.1), NS	10.5 (9.1–11.9)	1.3 (1.1–1.7), p = 0.02
**Employment status**	Paid employment	21.0 (18.1–23.9)	1.0	10.0 (8.9–11.2)	1.0
	Unemployed	26.2 (19.5–33.0)	1.2 (0.8–1.8), NS	25.1 (17.0–33.3)	1.7 (1.1–2.8), p = 0.03
	Economically inactive	12.3 (9.51–15.1)	0.6 (0.4–0.8), p = 0.001	5.7 (4.6–6.8)	1.1 (0.8–1.4), NS
**Housing tenure**	Own/mortgage	12.4 (9.4–15.4)	1.0	6.3 (5.5–7.2)	1.0
	Rented	20.1 (17.4–22.8)	1.4 (0.9–1.9), NS	15.3 (13.5–17.2)	1.8 (1.4–2.2), p<0.001
	Rent free	30.4 (20.8–39.9)	1.3 (0.8–2.3), NS	9.7 (4.7–18.8)	0.8 (0.3–1.9), NS

NS = non-significant; Weighted percentages to account for survey design;

a . Model adjusted for age and gender.

### Comparisons of CMD, hazardous alcohol use and illicit drug use in combined analysis

In Table 7 (full models presented in Table S1), the SELCoH sample was combined with data from the APMS England sample and the APMS sample from the Greater London area (N = 792) to determine whether or not differences in CMD, hazardous alcohol use and illicit drug use were explained by socio-demographic attributes and the socioeconomic status of the individual sample members. For the APMS London sample, the prevalence of CMD was 14.8% (95% CI 12.3–17.8), depressive episode was 2.6% (95% CI 1.7–4.1) and hazardous alcohol use was 22.6% (95% CI 19.4–26.3). Whereas, the prevalence of any drug use in the past year in the APMS London was greater than the national estimate and closer to the SELCoH sample estimate (APMS England = 8.9% (95%CI 8.1–9.8); APMS London = 13.1% (95%CI 9.9–17.2); SELCoH = 18.1% (95%CI 16.1–20.2). In the fully adjusted models, SELCoH participants had increased odds of CMD in comparison to both APMS England and APMS London participants after adjusting for socio-demographic and socio-economic indicators. SELCoH participants had lower odds of hazardous alcohol use than the APMS England and APMS London samples after adjusting for socio-demographic and socio-economic indicators. In contrast, SELCoH participants had more than twice the odds of reporting illicit drug use in the past year than the APMS England sample after adjusting for socio-demographic and socio-economic indicators in a combined dataset. This difference was weaker, but in the same direction in the comparison between the SELCoH sample and the APMS London sample in the fully adjusted model.

**Table 7:** Comparisons of adjusted odds ratios for common mental disorder, hazardous alcohol use and illicit drug use by socio-demographic and socio-economic indicators in combined data from both studies.

		Sample	Fully adjusted odds ratio^a^ (95%CI), p-value
**Common Mental Disorder**	**SELCoH and APMS England 2007**	SELCoH	1.4 (1.2–1.7), p<0.001
		APMS 2007	1.0
	**SELCoH and APMS London 2007**	SELCoH	1.7 (1.3–2.2), p<0.001
		APMS 2007	1.0
**Hazardous Alcohol Use**	**SELCoH and APMS England 2007**	SELCoH	0.8 (0.6–0.9), p = 0.001
		APMS 2007	1.0
	**SELCoH and APMS London 2007**	SELCoH	0.6 (0.4–0.8), p<0.001
		APMS 2007	1.0
**Illicit Drug Use**	**SELCoH and APMS England 2007**	SELCoH	2.1 (1.7–2.6), p<0.001
		APMS 2007	1.0
	**SELCoH and APMS London 2007**	SELCoH	1.6 (1.1–2.1), p = 0.008
		APMS 2007	1.0

NS = non-significant.

Weighted percentages to account for survey design.

a . Fully adjusted model with combined comparable data from both studies; age entered as a continuous variable in fully adjusted model.

## Discussion

### Main findings

In comparing two household psychiatric morbidity surveys from South East London and a national sample in England, this study addressed two aims: to compare (1) prevalence estimates of common mental disorder (CMD) in the past week, alcohol and illicit drug use in the past year and (2) whether or not any identified differences could be explained by socio-demographic attributes and the socioeconomic status of the individual sample members. There were several notable differences: the prevalence of CMD in the SELCoH sample was more than 10% higher than that in the national sample; there was a four-fold greater proportion of depressive episodes in the SELCoH sample; the national sample had a higher prevalence of hazardous alcohol use; and SELCoH participants had a higher prevalence of illicit drug use (individual drugs and poly drug use) in the past year. However, in both samples, men reported more hazardous alcohol and drug use than women, and cannabis was the most commonly reported drug followed by cocaine. Comparisons made in combined datasets showed that these differences between the SELCoH sample and the APMS England sample persisted after adjusting for socio-demographic and socioeconomic indicators for all outcomes. Further, similar differences were also present in comparisons between the SELCoH sample and the APMS subsample from the Greater London area in the fully adjusted models.

### Strengths and limitations

Among the strengths of this study are the similarities between the survey designs, the administration of the same validated structured clinical interview, and the representativeness of the APMS 2007 and the SELCoH samples. Specifically, the SELCoH sample was shown to be representative on most demographic and socioeconomic characteristics of the population in the study catchment area, the London boroughs of Southwark and Lambeth, according to the UK 2001 Census [16]. In terms of study limitations, non-response rates at the individual level in the national study and at the household level in the SELCoH sample may have resulted in participation bias, and the prevalence estimates should be considered with caution. A study linking a Norwegian household survey with disability pension registry data illustrated that mental and substance use disorders were strongly associated with non-participation, and conventional surveys may under-estimate prevalence of mental disorder [31]. Further, different weighting procedures were utilised and it is likely that there is residual confounding in the analysis examining differences between the two samples. In terms of the timing of the studies, APMS data were collected in the year leading up to the UK economic recession (2006–2007), while SELCoH data were collected after the recession period began (2008–2010). Common mental disorders, increased substance use and substance disorder are among the mental health problems more likely to increase during an economic downturn, primarily as a result of an increase in the poor socioeconomic conditions included in this analysis [32]–[34]. Finally, we acknowledge concerns about the validity of measures, such as the CIS-R [35]. However, as with the national study, we enlisted experienced and trained lay interviewers to administer the CIS-R. Given these strengths and limitations, it is worthwhile considering the extent to which differences in prevalence estimates may be real, or have resulted from the design and conduct of the two studies. Low participation rates may be a factor in any psychiatric epidemiological survey, and the lower participation rate in the SELCoH sample compared with the APMS sample may have led to an under-estimate of differences in prevalence estimates [31]. We used appropriate statistical methods to apply weights for each sample and to control for the effect of clustering by household within the SELCoH study in all analyses, so believe it unlikely that this contributed to the observed differences.

In theory these differences might have been a valid consequence of differences between the survey populations with respect to demographic profiles. The SELCoH sample was a somewhat younger and more female sample, with a much higher proportion of people with Black African or Caribbean ethnicity, and included more unemployed people and more manual workers, at the same time as having a better qualified population. Despite these differences in the demographic profile, there was a similar pattern of associations for the socio-demographic and socioeconomic indicators and the outcomes across the SELCoH and APMS samples. We argue that such differences in populations should not be seen as nuisance variables (i.e. confounders) but should be seen as plausible explanations for the differences observed. However, adjusting for these variables in analyses of combined datasets had no impact on the differences between the samples in frequency of disorders at the national and more local level. Thus, the differences are likely to be due to potential confounders not identified in this study. Further, it is notable that within virtually each stratum of the analyses presented, the prevalence of CMD and illicit drug use was higher and the prevalence of hazardous alcohol use was lower in the SELCoH sample than in the APMS sample.

### Comparisons with previous studies and implications

There were two main findings that deserve further discussion. First, to our knowledge there are no studies other than SELCoH that have compared UK inner city populations with national data in terms of prevalence of CMD or substance use. With the exception of more hazardous alcohol use in the national study, our findings support our hypotheses. Further, our findings are consistent with those from other national studies, such as the differences in CMD by urbanicity in the 1993 and 2000 British National Survey of Psychiatric Morbidity Surveys [11]–[12] and the graded increase in the prevalence of CMD and substance use disorders across multiple categories of urbanization in the Netherlands Mental Health Survey and Incidence Study [9]. Second, the findings on illicit drug use indicated that a focus on individual drugs, such as cannabis use and cocaine, are necessary, but poly drug use in the local community should also be of concern. In particular, simultaneous drug and alcohol use is associated with poor social outcomes and mental ill health in a US national household survey [28]. More broadly, explanations for the differences in substance use between samples are likely to be rooted in the social environment. An international review of the social epidemiology of substance use suggested that type and density of social networks and neighbourhood-level indicators were associated with increased substance use, whereas findings on the relationship between socioeconomic status (SES) and substance use were mixed [36]. The latter is highlighted in further national and local comparisons; high SES groups were more likely to report hazardous alcohol use in the SELCoH sample, whereas there were no differences by SES in the APMS or the British National Survey of Psychiatric Morbidity 2000 [37]. Further investigations of social and environmental factors that influence the population distribution of CMD and substance use are needed to better understand these differences between and within national and local community samples.

The findings of this study highlight the importance of considering differences between local and national public mental health that are needed to inform service provision and policy, specifically regarding the distribution of resources. While there are similarities in the social inequalities present at national and local levels that greatly contribute to the generation of mental health inequalities, differences in the prevalence of CMD and drug use justify the need to consider specific public mental health needs of those residing in urban environments. The findings resulting from comparisons between the SELCoH sample and the APMS London sample further highlight the need to drill down from the metropolitan area to the local area level. The growing number of individuals moving into urban environments, the impact of social relationships [36], [38] and the urban context on health [39]–[40] suggests that there is a growing need for locally focused public mental health studies. A practical solution to this problem may be as simple as a joint investment by local and national public health and service provision authorities to collect comparable data at the local level in combination with monitoring inequalities by using secondary data sources such as electronic health records and case registers.

## Supporting Information

Table S1 **Comparisons of adjusted odds ratios for illicit drug use by socio-demographic and socio-economic indicators combined data from both studies (full models).**
(DOCX)
