# Supplementary material for: Importance of Thinking Locally for Mental Health: Data from Cross-Sectional Surveys Representing South East London and England
Source: PLoS One. 2012 Dec 12;7(12):e48012. doi: 10.1371/journal.pone.0048012 (PMC3520993; doi:10.1371/journal.pone.0048012)
Supplement: Table S1 — Comparisons of adjusted odds ratios for illicit drug use by socio-demographic and socio-economic indicators combined data from both studies (full models). (DOCX) [file pone.0048012.s001.docx]

Table S1 Comparisons of adjusted odds ratios for illicit drug use by socio-demographic and socio-economic indicators combined data from both studies (full models)

|  |  | **CMD** |  | **Hazardous Alcohol Use** |  | **Illicit Drug Use** |  |
| --- | --- | --- | --- | --- | --- | --- | --- |
|  |  | **SELCoH and APMS England 2007** | **SELCoH and APMS London 2007** | **SELCoH and APMS England 2007** | **SELCoH and APMS London 2007** | **SELCoH and APMS England 2007** | **SELCoH and APMS London 2007** |
|  |  | Fully adjusted odds ratio^a^ (95%CI), p-value | Fully adjusted odds ratio^a^ (95%CI), p-value | Fully adjusted odds ratio^a^ (95%CI), p-value | Fully adjusted odds ratio^a^ (95%CI), p-value | Fully adjusted odds ratio^a^ (95%CI), p-value | Fully adjusted odds ratio^a^ (95%CI), p-value |
| **Sample** | SELCoH | 1.4 (1.2 - 1.7), p<0.001 | 1.7 (1.3 – 2.2), p<0.001 | 0.8 (0.6 - 0.9), p=0.001 | 0.6 (0.4 - 0.8), p<0.001 | 2.1 (1.7 - 2.6), p<0.001 | 1.6 (1.1 - 2.1), p=0.008 |
|  | APMS 2007 | 1.0 | 1.0 | 1.0 | 1.0 | 1.0 | 1.0 |
| **Gender** | Female | 1.6 (1.4 - 1.9), p<0.001 | 1.8 (1.4 – 2.2), p<0.001 | 0.4 (0.3 - 0.4) p<0.001 | 0.3 (0.3 - 0.4) p<0.001 | 0.5 (0.4 - 0.6), p<0.001 | 0.5 (0.3 - 0.6), p<0.001 |
|  | Male | 1.0 | 1.0 | 1.0 | 1.0 | 1.0 | 1.0 |
| **Ethnic group** | White British | 1.0 | 1.0 | 1.0 | 1.0 | 1.0 | 1.0 |
|  | Black  Caribbean | 1.1 (0.7 - 1.5), NS | 1.2 (0.8 - 1.8), NS | 0.3 (0.2 - 0.5) p<0.001 | 0.3 (0.1 - 0.5) p<0.001 | 1.2 (0.8 - 1.9), NS | 0.9 (0.6 - 1.5), NS |
|  | Black African | 0.8 (0.6 - 1.1), NS | 0.8 (0.6 - 1.2), NS | 0.1 (0.1 - 0.2) p<0.001 | 0.1 (0.1 - 0.2) p<0.001 | 0.2 (0.1 - 0.4), p<0.001 | 0.2 (0.8 - 0.3), p<0.001 |
|  | Asian | 0.9 (0.6 - 1.2), NS | 1.2 (0.7 - 1.9), NS | 0.2 (0.1 - 0.3) p<0.001 | 0.2 (0.1 - 0.4) p<0.001 | 0.2 (0.1 - 0.4), p<0.001 | 0.2 (0.1 - 0.4), p<0.001 |
|  | Other | 1.0 (0.8 - 1.2), NS | 0.9 (0.7 - 1.4), NS | 0.6 (0.5 - 0.8), p=0.001 | 0.5 (0.3 - 0.7), p=0.001 | 1.0 (0.7 - 1.4), NS | 0.8 (0.6 - 1.2), NS |
| **Age (years) ^b^** |  | 0.9 (0.9 - 0.9), p<0.001 | 0.9 (0.9 -1.0), NS | 0.9 (0.9 - 0.9), p<0.001 | 0.9 (0.9 - 0.9), p<0.001 | 0.9 (0.9 - 0.9), p<0.001 | 0.9 (0.9 - 0.9), p<0.001 |
| **Marital status** | Never married | 1.0 | 1.0 | 1.0 | 1.0 | 1.0 | 1.0 |
|  | Married or cohabiting | 0.9 (0.8 -1.1), p<0.001 | 0.7 (0.5 -0.9), p=0.01 | 0.7 (0.6 - 0.8), p<0.001 | 0.7 (0.5 - 0.9), p=0.004 | 0.5 (0.4 - 0.6), p<0.001 | 0.4 (0.3 – 0.6), p<0.001 |
|  | Divorced or separated | 1.7 (1.4 - 2.1), p<0.001 | 1.2 (0.8 – 1.7), NS | 1.0 (0.8 - 1.2), NS | 1.3 (0.8 - 1.9), NS | 1.3 (0.9 - 1.8), NS | 1.1 (0.7 - 1.8), NS |
|  | Widowed | 1.1 (0.8 -1.5), NS | 1.1 (0.6 -2.0), NS | 0.6 (0.4 - 0.8), p=0.002 | 0.6 (0.2 – 1.7), NS | 0.8 (0.4 - 1.6), NS | 0.4 (0.1 - 1.5), NS |
| **Education levels** | No qualifications | 1.4 (1.2 - 1.8), p<0.001 | 1.5 (1.0 – 2.1), p=0.04 | 0.9 (0.7 - 1.0), NS | 0.7 (0.4 - 1.1), NS | 0.9 (0.7 - 1.3), NS | 0.9 (0.6 - 1.5), NS |
|  | Up to GCSE level | 1.3 (1.1 - 1.6), p=0.001 | 1.5 (1.1 – 2.1), p=0.004 | 0.7 (0.6 - 0.8), p<0.001 | 0.4 (0.3 - 0.6), p<0.001 | 0.8 (0.6 - 1.0), NS | 0.8 (0.5 - 1.2), NS |
|  | Advanced level | 1.2 (0.9 - 1.4), NS | 1.3 (0.9 - 1.8), NS | 0.9 (0.8 - 1.1), NS | 0.7 (0.6 - 1.1), NS | 0.9 (0.7 - 1.2), NS | 1.0 (0.7 - 1.5), NS |
|  | Higher degree or above | 1.0 | 1.0 | 1.0 | 1.0 | 1.0 | 1.0 |
| **Employment status^c^** | Paid employment | 1.0 | 1.0 | 1.0 | 1.0 | 1.0 | 1.0 |
|  | Unemployed | 1.8 (1.3 - 2.5), p<0.001 | 2.0 (1.3 – 3.1), p=0.002 | 1.1 (0.7 - 1.5), NS | 1.1 (0.7 - 1.9), NS | 1.3 (0.9 – 1.9), NS | 1.1 (0.7 – 1.7), NS |
|  | Economically inactive | 1.4 (1.2 – 1.6), p<0.001 | 1.2 (0.9 – 1.6), NS | 0.8 (0.7 - 0.9), p=001 | 0.9 (0.6 -1.2), NS | 0.7 (0.6 – 0.9), p=0.01 | 0.6 (0.4 – 0.8), p=0.001 |
| **Housing tenure** | Own/mortgage | 1.0 | 1.0 | 1.0 | 1.0 | 1.0 | 1.0 |
|  | Rented | 1.6 (1.4 – 1.8), p<0.001 | 1.3 (0.9 – 1.7), NS | 1.1 (0.9 – 1.3), NS | 0.9 (0.6 – 1.2), NS | 1.6 (1.3 – 2.0), p<0.001 | 1.4 (0.9 – 1.9), NS |
|  | Rent free | 0.9 (0.6 - 1.4), NS | 0.6 (0.3 - 1.1), NS | 1.2 (0.8 – 1.9), NS | 1.0 (0.6 – 1.8), NS | 1.0 (0.6 – 1.6), NS | 1.1 (0.6 – 2.0), NS |

NS=non-significant

Weighted percentages to account for survey design.

a. Fully adjusted model with combined data from both studies

b. Age entered as a continuous variable in fully adjusted model

c. Social class excluded from fully adjusted models due to missing values in SELCoH
